# Perspective: thermo-sensation to thermoregulation: advancing the anticipation debate via transient receptor potential (TRP) channels

**DOI:** 10.1007/s00421-026-06228-3

**Published:** 2026-04-06

**Authors:** Frank E. Marino

**Affiliations:** School of Rural Medicine, Charles Sturt University, Leeds Parade, Orange, NSW 2800 Australia

**Keywords:** Thermoregulation, TRPV1, Anticipatory regulation, Pacing, Heat acclimation, Group III/IV afferents

## Abstract

**Purpose:**

To revisit the anticipatory regulation model of exercise in heat and show how contemporary molecular evidence, particularly transient receptor potential (TRP) channels provide a plausible mechanistic basis for trajectory-sensitive regulation beyond than classic cellular catastrophe or limitation paradigm.

**Approach:**

By synthesising self-paced and fixed-rating of perceived exertion (RPE) findings with recent molecular and integrative physiology, it proposes a closed-loop framework where thermal signals from muscle/skin are transduced by TRPs and integrated centrally to shape pacing in real time.

**Evidence:**

Evidence consistent with anticipatory regulation includes earlier environment-sensitive reductions in power and skeletal muscle electromyography before lethal core temperature divergence, RPE-clamp profiles that align subsequent heat storage across conditions, and field data showing pace selection that preserves safe heat-storage trajectories. At the molecular level, TRPV1 in skeletal-muscle sarcoplasmic reticulum links rising local temperature to Ca²⁺, signals then downstream leading to PGC-1α signalling promoting mitochondrial biogenesis and endurance capacity, while group III/IV afferents convey thermal/metabolic state to the central nervous system. Warm-sensitive TRPs in hypothalamic circuits operate near physiological brain temperature and contribute to thermoeffector drive, completing a periphery - central feedforward/feedback loop.

**Conclusions:**

A thermo-TRP closed loop framework can reconcile “integrated strain” and anticipatory models as different descriptions of the same system while clarifying the boundary between evidence and biological plausibility when extrapolating from molecular studies to whole body regulation. This framework yields testable predictions and clarifies why interventions such as skin cooling and heat acclimation improve safety margins by altering thermal trajectory, not just peak core temperature.

## Introduction

Two decades ago, the anticipatory regulation and avoidance-of-catastrophe model was proposed as an alternative to fixed “critical core temperature” explanations of fatigue (Marino 2004). Rather than shutting down at ~ 40 °C, it was hypothesised that the central nervous system (CNS) modulates work output according to the *rate* at which temperature rises, so exercise terminates before hyperpyrexia ensues (Marino et al. 2004b; Marino 2004). The earliest explicit pushback to this proposition followed soon after which argued that the classic cardiovascular and metabolic constraints are sufficient to explain fatigue and that the central-governor/anticipatory model lacked convincing support (Weir et al. 2006), citing oxygen consumption (VO₂) and cardiac output limitations and the absence of robust evidence for key predictions, a view amplified by empirical attempts (Brink-Elfegoun et al. 2007a, b; Shephard 2009). Advancing this debate now turns on molecular signalling. Identifying mechanisms that transduce rising tissue temperature into rapid ionic and afferent signals which provide the avenue and footing that the anticipatory model was once said to lack.

“Anticipatory regulation” relates to a trajectory-management strategy where an athlete sets an initial pacing plan from endpoint knowledge and prior experience (feedforward), then continuously updates that plan using weighted sensory feedback about the current thermo-metabolic state (feedback). In this framing, “anticipation” relates managing the rate of heat storage rather than defending a fixed core temperature (T_c_) threshold. For clarity, if links are demonstrated directly they are described as evidence, whereas, links inferred from molecular or animal work are labelled as biological plausibility or hypotheses (see Fig. 2 legend).

However, this debate is not merely semantic; it reflects competing paradigms grounded in different observations. The conventional view, by emphasizing cardiovascular and metabolic ceilings, implicitly introduced the language of “catastrophe” or *limitation* into exercise physiology (Noakes et al. 2004; Noakes 2011). In practice, however, skeletal muscle cannot be driven to rigor in intact humans except under pathological/poisoning conditions; protective mechanisms curtail contraction before cellular collapse (Edwards 1983). A core feature of catastrophe theory across disciplines (biology, engineering etc.) is that when equilibrium breaks down, catastrophe ensues, but, importantly, hysteresis (recovery after the threat is averted) is the rule rather than the exception (Zeeman 1976; Edwards 1983).

In the specific context of exercise in the heat, critics argued that reduced power output is sufficiently explained by the integrated burden of thermal, cardiovascular and perceptual strain, with self-paced efforts maintained at a similar relative intensity until accumulating physiological strain necessitates pacing adjustments (Weir et al. 2006; González-Alonso 2007; Nybo 2008; Périard et al. 2022). They further suggested that end-spurts reflect knowledge of the endpoint and anticipated work remaining rather than the sudden release of a centrally held “reserve” (St Clair Gibson et al. 2006; Abbiss and Laursen 2008). A broader critique concluded the “central governor” family of exercise physiology models lacked robust experimental support, pointing instead to ceilings in oxygen transport and clear cardiovascular/thermoregulatory constraints as adequate explanations (Joyner and Coyle 2008; Shephard 2009). From a conceptual point of view, exercise is regulated in the heat as a feedforward/feedback loop in which predictive drive (endpoint knowledge, prior state) is continuously calibrated by weighted thermal feedback.

Accordingly, the purpose of this Perspective is to comment on how earlier physiological models, particularly anticipatory regulation, can now be reconciled with contemporary molecular and integrative evidence, and to outline testable predictions that follow.

## Signatures of anticipation: power, electromyography, and heat storage

A recent field editorial added a vivid *N* = 1 case in which, during an unacclimatised desert time trial, an initially optimistic pace collapsed, T_c_ exceeded 41 °C, with seemingly poor anticipation (Nybo 2025). While such field narratives are valuable for hypothesis generation, single-participant observations cannot resolve mechanistic workings and are particularly sensitive to context (hydration status, airflow, pacing intent, measurement error). Rather than falsifying anticipation, such examples are compatible with a control system whose feedback is delayed and/or whose gain changes with state.

Against this backdrop, it is important to recognise that we (Kay et al. 2001; Marino et al. 2004a) and others (Tucker et al. 2004, 2006; Swart et al. 2009) reported findings consistent with anticipatory regulation, although these observations are not simply explained by that model. In prolonged self-paced exercise in the heat, progressive cardiovascular strain, including reductions in stroke volume, cardiac output and peak oxygen uptake as thermal load increase which can also attenuate power output (Périard et al. 2011). Similarly, when comparing African and Caucasian runners of different stature, some of the differences in heat storage trajectory potentially reflected metabolic heat production relative mass and body size where heat exchange characteristics relative exercise intensity may not have been normalised (Cramer and Jay 2014). Accordingly, these earlier findings are likely better interpreted as compatible with, rather than definitive proof of, anticipatory regulation. Even so, in 35 °C versus 15 °C self-paced cycling, power and vastus lateralis electromyographic (EMG) signals fell earlier and further despite similar Tc for most of the trial, indicating that skeletal muscle recruitment declined before excessive T_c_ developed (Tucker et al. 2004). Under a rating of perceived exertion (RPE) clamp protocol, an initially steeper rate of heat storage in 35 °C was followed by a linear down-regulation of power such that subsequent heat storage matched cooler conditions (Tucker et al. 2006). When running in the heat, smaller African runners maintained higher speeds yet finished with similar terminal T_c_ to their heavier Caucasian counterparts, a pattern compatible with pace selection from the outset to keep the trajectory of heat storage within safe bounds, while acknowledging that differences in relative heat production may also have contributed (Marino et al. 2004a). Related analyses and rebuttals explain why end-spurts, early EMG divergence and submaximal activation at exhaustion remain difficult to reconcile with purely peripheral “catastrophe” accounts (Noakes and Marino 2007, 2008).

## A mechanistic bridge: from strain to signals

**Fig. 1:**
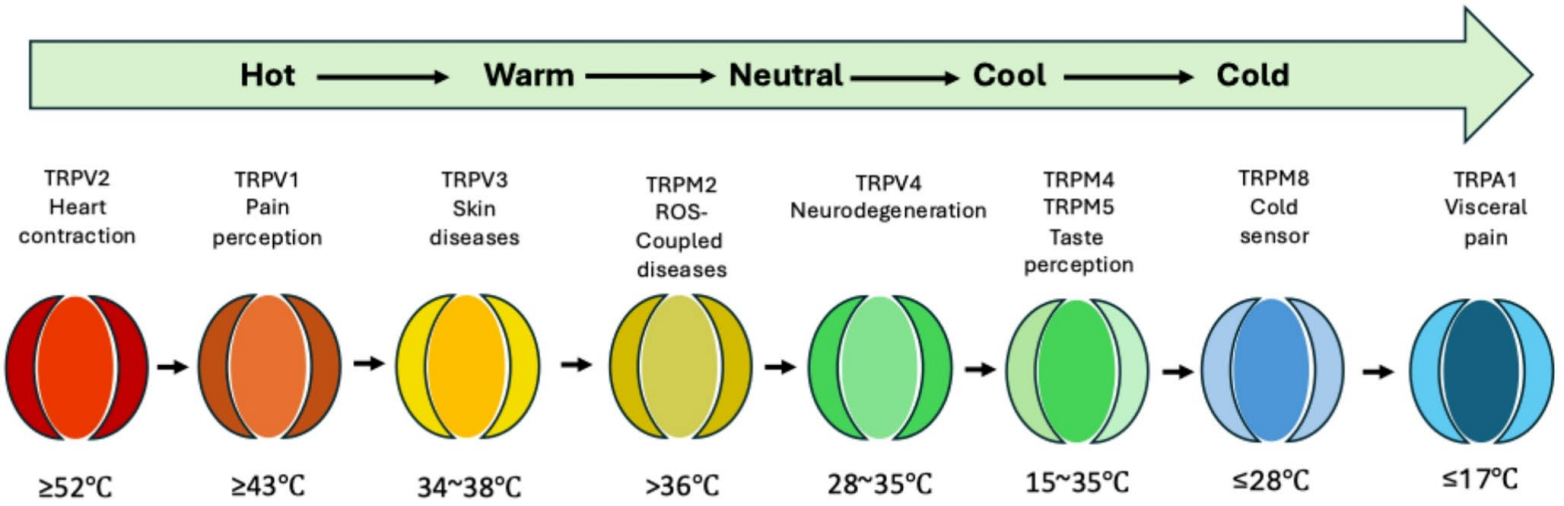
Schematic overview of major mammalian TRP channels and their approximate activation ranges across the thermal continuum. Canonical heat-sensitive channels (e.g., TRPV1, TRPV2, TRPV3, TRPM2, TRPV4) and cool/cold sensors (TRPM8, TRPA1) are shown with representative thresholds. Thresholds vary by preparation and are shifted by the local milieu (e.g. pH, phosphorylation, lipids/ligands, redox), so values should be interpreted as context dependent. Figure redrawn and adapted from Yan et al. (2022) *Frontiers in Pharmacology* CC-BY license

**Fig. 2:**
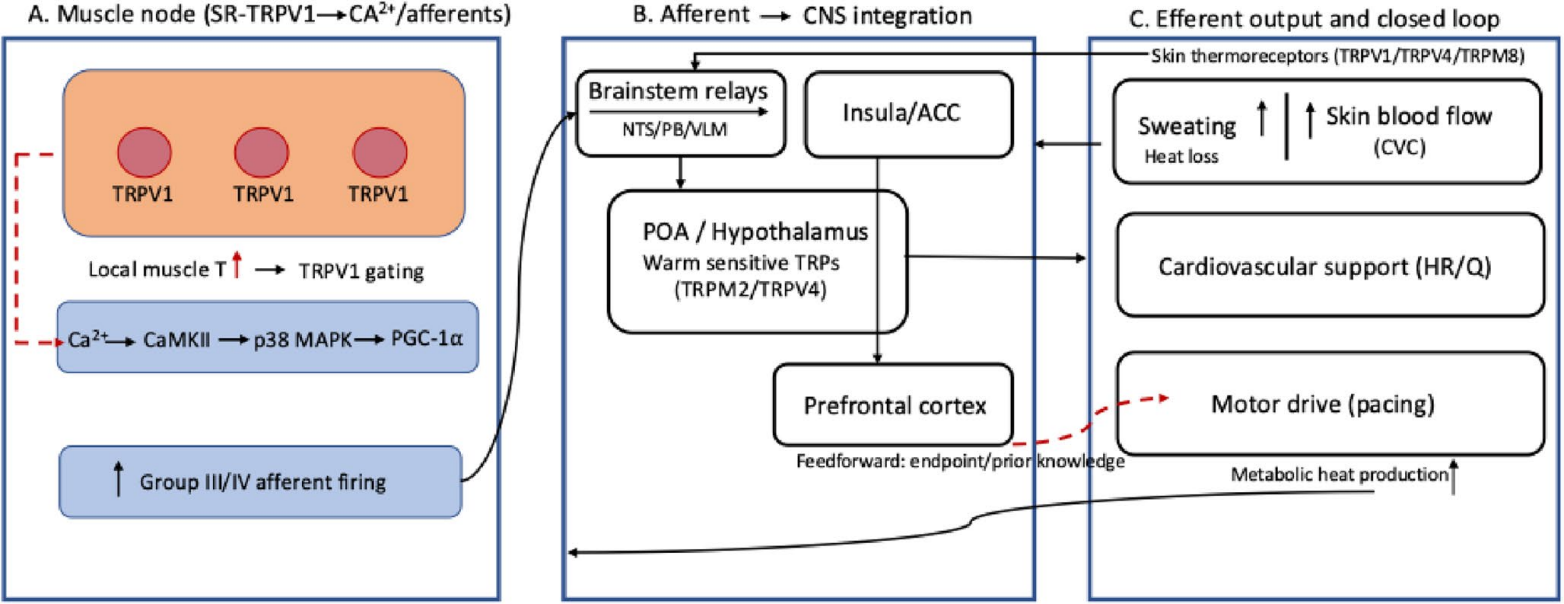
Proposed feedforward/feedback framework for exercise in the heat (conceptual). Solid arrows denote links that are well supported (e.g., thermo-TRP expression in sensory pathways; hypothalamic integration of thermal input); dashed arrows denote hypothesised links or links supported primarily by animal/cell work (e.g., SR-TRPV1–Ca²⁺–PGC-1α signalling in skeletal muscle; potential contribution of TRP-weighted afferent input to online pacing adjustments). Local warming and local milieu are proposed to increase the open probability of SR-localised TRPV1, generating Ca²⁺ signals that can (i) engage CaMKII–p38 MAPK–PGC-1α programmes (training adaptation) and (ii) contribute via group III/IV afferent traffic and perception to feedback that can bias motor output. Afferent signals ascend via spinal/brainstem relays (NTS/PB/VLM) to the preoptic area (POA)/hypothalamus, where warm-sensitive populations (including TRPM2/TRPV4-expressing neurons) and pyrogenic inputs (PGE₂→EP3) shape thermoeffector drive and interact with insula/ACC and prefrontal cortex (task context/decision-making). Descending commands adjust sweating, skin blood flow, cardiovascular responses and motor drive (pacing), altering heat loss and metabolic heat production and feeding back to muscle and skin sensors. In this framework, a feedforward pacing plan (endpoint/prior knowledge) is continuously calibrated by weighted sensory feedback, so effort is regulated to the trajectory of heat storage (e.g., rate of change in T_c_) rather than a single threshold. *ACC* anterior cingulate cortex, *CVC* cutaneous vascular conductance, *NTS* nucleus tractus solitarius, *POA* preoptic hypothalamus, *PB* parabrachial nucleus, *VLM* ventrolateral medulla, *PGE₂→EP3* prostaglandin E2 binding to the EP3 receptor, *HR* heart rate, *Q* cardiac output

What has changed since the original debate is the molecular background. The transient receptor potential (TRP) channels, now recognised in mammals as polymodal sensors of temperature and chemicals, provide a plausible substrate, which, by their presence and function integrate the periphery with the CNS (Vriens et al. 2014). The mammalian “thermo-TRP” era was crystallised with the cloning of TRPV1 as the capsaicin/heat receptor (Caterina et al. 1997). This was followed by identification of TRPM8 as the principal detector of environmental cold (McKemy et al. 2002; Peier et al. 2002). Genetically, TRPM8-null mice show marked behavioural deficits to cold, while human TRPV1 antagonists were shown to have reproducible, concentration-dependent hyperthermia, consistent with a tonic contribution of TRPV1-linked pathways to body temperature control. Thermo-TRP activation ranges are summarised in Fig. 1; critically, these operating ranges are plastic so that pH, phosphorylation and lipid mediators can sensitise heat-gated TRPs (lowering thresholds), whereas acclimation and chaperone induction may reduce system gain (Rosenbaum and Simon 2007; Périard et al. 2015; Nava and Zuhl 2020). Equally important for the closed-loop model (Fig. 2), TRPV1 signalling is unlikely to scale linearly with temperature. As suggested by the activation range depicted in Fig. 2, TRPV1 may behave more like a threshold sensor rising sharply once local temperatures reach its operating range and then flattening with sustained activation, such that TRPV1 dependent input to afferent and intracellular pathways becomes less discriminating at higher, prolonged heat loads (Fig. 2) (Mohapatra and Nau 2005; Rosenbaum and Simon 2007; Vyklický et al. 2008).

Together, these discoveries moved thermosensation from a black box to identified molecular pathways that can link peripheral and central sensors. In relation to exercise physiology, they can advance testable predictions about how rising skeletal muscle and skin temperature may be converted into signals that contribute to pacing adjustments, perception, and thermoeffector drive.

In practical terms, the “integrated burden” of heat stress only becomes actionable when it is converted into ionic and chemical signals the nervous system can interpret. Thermosensitive TRP channels are one class of transducer that can contribute to this conversion, alongside other thermal, metabolic and mechanosensitive receptors. Accordingly, Fig. 2 presents a closed loop framework in which TRP-linked inputs participate in coupling peripheral heat production to central integration and effector output.

Peripherally, functional TRPV1 has been localised to the sarcoplasmic reticulum (SR) in rodent skeletal muscle fibres, where heat or agonists can evoke Ca²⁺ release and leakage into the cytosol (Lotteau et al. 2013). These Ca²⁺ signals can engage CaMKII and downstream p38 MAPK pathways that converge on PGC-1α, a key regulator of oxidative mitochondrial remodelling (Luo et al. 2012; Xu et al. 2022). This molecular pathway provides a route by which repeated thermal exposure during physical training could contribute to endurance adaptation. However, direct evidence that SR-TRPV1 signalling acutely regulates moment-to-moment pacing in humans is currently limited; here it is used as biological plausibility for how local heat can be transduced into signals that, in principle, could inform a closed-loop regulator (Luo et al. 2012; Xu et al. 2022).

The study by Luo et al. (2012) is particularly relevant to the present argument. These authors considered whether activating TRPV1 drives endurance-type adaptations in skeletal muscle. They combined in vitro work (C2C12 myotubes) with mouse experiments using dietary capsaicin, a known TRPV1 agonist with TRPV1 overexpression, and TRPV1-knockout controls. In myotubes, capsaicin increased cytosolic Ca²⁺ and upregulated PGC-1α. In vivo, capsaicin feeding (and TRPV1 overexpression) increased PGC-1α, boosted genes for mitochondrial respiration and fatty-acid oxidation, shifted skeletal muscle fibres toward a more oxidative profile, and improved exercise endurance; these effects were absent in knock-out mice. Thus, TRPV1 can translate local chemical and thermal stimuli into durable, performance relevant adaptations. Importantly, this evidence relates most directly to training adaptation rather than acute pacing modulation, with the work relied on being a mouse model with systemic capsaicin exposure (with potential peripheral and central effects). Accordingly, in this Perspective, the Luo et al. pathway is used to support mechanistic plausibility for a peripheral “strain-to-signal” transduction step, rather than proof that TRPV1 acts as a real time controller of human work output.

The same thermal and metabolic milieu increases the discharge of group III/IV afferents, exporting a real-time “strain signal” to the spinal cord and brainstem (Rotto and Kaufman 1988; McCord and Kaufman 2009). In most preparations, afferent firing reflects a convergence of inputs which include temperature, mechanical distortion, and metabolites such as pH, ATP and inflammatory mediators, rather than a purely thermal signal. Accordingly, TRP channels are not proposed to account for all afferent traffic. Rather, TRP linked heat sensitivity could contribute directly or indirectly by shaping local Ca²⁺ handling, metabolite accumulation and inflammatory signalling that modulate afferent excitability.

Centrally, warm-sensitive neurons in the preoptic/anterior hypothalamus operate in the physiological warm range and include populations defined by TRP expression (notably TRPM2 and TRPV4), integrating deep-brain temperature with viscero-somatic and pyrogenic input to shape thermo-effector drive (Lazarus et al. 2007; Song et al. 2016; Tan and Knight 2018; Shibasaki 2024) (Vriens et al. 2014). These hypothalamic integrators interact with interoceptive and decision related networks (e.g., insula and anterior cingulate cortex, Fig. 2), providing a plausible route by which thermal state can influence perceived strain and, indirectly, voluntary work rate with adjustments in skin blood flow, sweating, heart rate and motoneuron drive. In acclimatised states, expanded plasma volume and heat shock protein (HSP) induction likely shift TRP operating points and flatten feedback gain (Nava and Zuhl 2020). As such, “integrated strain” is not an alternative to anticipatory control but its molecular substrate. The TRP-mediated signalling supplies early, graded information that lets the CNS regulate effort according to the “trajectory” of heat storage, precisely the behaviour observed and reported in self-paced (Kay et al. 2001; Marino et al. 2004a; Wingfield et al. 2018) and RPE-clamp experiments (Tucker et al. 2004, 2006; Flood et al. 2017).

## Re-reading classic objections to anticipatory regulation through a TRP lens

The principal criticism of anticipatory regulation paradigm was that reductions in power during exercise in the heat could in part be explained by the integrated burden of thermal, cardiovascular and perceptual strain, without requiring a distinct anticipatory controller. This systems level description indeed captures important constraints on performance. The point here is narrower; that is, for such strain to influence behaviour, it must be *transduced* or *converted* into neural information that can be integrated and acted upon. In this context, TRP channels are not proposed as controllers of work output, but as molecular transducers which might contribute to the afferent information flow with other thermal, metabolic and mechanosensitive receptors.

In rodent skeletal muscle, evidence indicates that SR-localised TRPV1 can couple local heat and chemistry to Ca²⁺ signalling and downstream remodelling pathways (Luo et al. 2012; Lotteau et al. 2013; Xu et al. 2022). In sensory pathways, thermo-TRPs and other receptors contribute to afferent traffic informing brainstem, hypothalamic and perceptual integration. Accordingly, what has been described as “integrated strain” can be viewed as the systems level consequence of multiple convergent inputs, of which TRP linked signalling may be one component rather than the sole substrate. Framed this way, TRP biology supplies a mechanistic layer beneath the integrated strain description and generates testable predictions about when feedback arrives early versus late, or with high versus low gain, without claiming that TRP signalling alone explains all variance in pacing.

A second objection was that the central governor (CG)/anticipatory regulation models were unnecessary because classic ceilings in oxygen transport and thermoregulation are sufficient to explain task failure. Those ceilings are not disputed here; rather, the question is how the organism navigates the available operating space as heat storage evolves. TRP linked transduction provides a plausible means by which local thermal state and its interaction with the chemical milieu, could be converted into afferent traffic and perceptual signals that arrive early enough to bias autonomic and motor output well before ceilings are breached. This reframes the anticipatory regulation/CG debate from a binary (“exists/doesn’t exist”) into a feedback-timing and gain problem. That being, when TRP-weighted signals arrive early (e.g., acclimatised, high skin blood flow, lower TRPV1 gain), pacing drifts gradually but when they arrive late or are high-gain (e.g., unacclimatised, sensitised TRPV1), pacing can appear “catastrophic.” The observation of submaximal EMG at task failure and environment-sensitive early reductions in power is naturally predicted by such closed-loop control, without denying oxygen-transport limits (Tucker et al. 2004, 2006).

A further objection was that end-spurts reflect endpoint knowledge and motivation rather than a hidden physiological reserve. The TRP view is compatible with this literature. Knowledge of the endpoint sets the reference trajectory for energy distribution (Foster et al. 2004; St Clair Gibson et al. 2006; Abbiss and Laursen 2008). TRP-weighted feedback simply bounds that plan so that as peripheral SR-TRPV1 activity and central warm-sensor firing indicate safe margin near the finish (cooler skin, increased convective loss, declining heat storage), the controller permits upregulation of effort.

Finally, the overshoot episodes (e.g., unacclimatised desert time trial) are sometimes interpreted as failures of anticipation (Nybo 2025). However, the TRP framework predicts context-dependent gain and delay. In unacclimatised states, sensitised TRPV1 and limited skin blood flow raise local muscle temperature faster for a given power simultaneously, central integration may prioritise core over skin signals early in heat exposure. The result is a late, high-amplitude correction which leads to dramatic power collapse with very high core temperature, yet still short of cellular catastrophe because the loop eventually curtails motor drive. Conversely, acclimation related adaptations (e.g. plasma volume expansion, altered skin/core weighting, HSP induction and possible shifts in TRP operating) might reduce the effective gain and/or delay, producing steady pacing even when end of exercise T_c_ remains elevated.

In summary, re-reading earlier objections through a TRP lens suggests that classic “integrated strain” and anticipatory frameworks may describe the same system at different levels of analysis. TRP channels (e.g., TRPV1, TRPM8, TRPM2/TRPV4) provide molecular candidates for transducing thermal and chemical state into afferent and intracellular signals, but the extent to which these pathways acutely modulate human pacing remains an open empirical question. The value of the present synthesis is, therefore, not to “resolve” the debate, but to sharpen mechanistic plausibility, specify boundary conditions (gain, delay, non-linearity), and articulate operational predictions about how perturbing skin and muscle thermal inputs or system state (acclimation) could reshape heat-storage trajectories and pacing.

## Testable predictions

The following predictions are framed to be experimentally operational and falsifiable, and to distinguish acute pacing modulation from training adaptation.

For instance, an acute local muscle-temperature perturbation could be tested using crossover designs during a self-paced time trial (or an RPE-clamp protocol) performed in the heat. In one condition acute local muscle-temperature perturbation could be tested with the working quadriceps selectively warmed by approximately 1–2 °C, while whole-body T_c_ core T_sk_ are matched. The primary endpoints would be early power output, EMG activity, ratings of thermal sensation and discomfort, and the T_c_ trajectory across the exercise bout. The prediction is that local quadriceps warming will shift the pacing trajectory toward earlier down-regulation (or require lower power to maintain the same RPE), accompanied by a steeper early rise in T_c_. This prediction would be falsified if pacing behaviour, EMG, and the T_c_ trajectory are unchanged despite the local muscle warming. Therefore, by selectively elevating local quadriceps temperature while matching T_c_ and T_sk_, this design selectively tests the muscle-local thermal input as shown in Fig. 2, allowing us to test whether deep thermos-sensory drive measurably reshapes the pacing trajectory. A shift toward earlier power downregulation (or lower power at a fixed RPE) under these conditions would be consistent with a TRP-weighted contribution to the moment-by-moment strain signal, whereas no change would argue against a meaningful role for local muscle temperature input in real time pacing under heat stress.

An additional possibility is to test the acute skin – core dissociation by using a water-perfused suit or fan and misting to manipulate T_sk_ (cool vs. warm) while clamping metabolic rate (or workload) and monitoring T_c_. The primary endpoints would be power output or pace, perceived thermal strain, sweating and skin blood flow, and the T_c_ trajectory across the exercise bout. The prediction is that cooler skin at a matched T_c_ will permit higher early power or a larger end-spurt, whereas warmer skin will bring forward pacing reductions and elevate perceived thermal strain. This prediction would be falsified if pacing remains invariant to T_sk_ manipulation when T_c_ and workload are matched. Therefore, in the TRP based framework (Fig. 2), this protocol selectively manipulates the cutaneous thermal sensation end of the loop, where changing T_sk_ alters TRP mediated skin afferent traffic (and therefore thermal sensation/discomfort) without requiring a change in T_c_, which is exactly the kind of “sensory reweighting” the model predicts can reshape pacing. If cooler skin permits higher early power or a larger end-spurt at matched T_c_ and workload, that would be consistent with a TRP weighted contribution of skin derived thermal input to the real time integrated strain estimate. That is, if pacing is unchanged despite large T_sk_ driven shifts in perceived thermal strain and thermos-effector responses, it would argue that skin TRP input is not a meaningful driver of pacing under those constrained conditions or is overridden by other signals.

## Implications

This perspective clarifies when and why interventions work by showing how they modulate the thermal signals that shape pacing. For athletes and heat-exposed workers, skin-focused cooling such as fans, misting, ice towels, conductive or evaporative garments, helps not only through small changes in T_c_ but by down-weighting skin-derived TRP input. The result is safer early pacing for the same T_c_ trajectory. By contrast, pre-event heating that potentiates heat-sensitive pathways may steepen early thermal discomfort and bring forward pacing reductions; whether that is useful depends on context and is not assumed to be uniformly beneficial in the heat.

Heat acclimation remains the foundation as it expands plasma volume, improves skin blood flow and sweating, induces heat-shock proteins, and can alter sensory weighting. Within the Fig. 2 closed-loop framing, acclimation is hypothesised to reduce the effective gain linking a given change in thermal inputs (T_c_, T_sk_) to changes in perceived strain and, in turn, pacing. In practical terms, the same perturbation in thermal inputs would evoke a smaller rise in perceived thermal strain and a smaller pacing adjustment, which could promote steadier pacing and a larger physiological ‘buffer’ even when terminal T_c_ is high.

For coaching and monitoring, it would be prudent to move beyond single endpoints to trajectory metrics. Tracking the rate of rise in T_c_ and cumulative heat storage, so that session targets are set around these, not just a peak T_c_ might be more practical. In clinical and occupational settings, a TRP lens helps explain exertional heat illness risk (high-gain feedback loops or delayed signals), symptoms in small-fibre neuropathies (altered TRP signalling), and drug effects such as hyperthermia with TRPV1 antagonists. Screening by phenotype (strong early thermal discomfort) or genotype (TRP variants) is speculative at present and would require prospective validation. A more immediate implication is to individualise cooling and work – rest strategies for those who show disproportionate thermal discomfort or rapid heat-storage trajectories.

Identification of thermo-TRP channels in skeletal muscle and brain reveals the molecular architecture for trajectory-sensitive feedback that couples heat production to autonomic and behavioural outputs. This “hardware” strengthens mechanistic plausibility for anticipatory regulation and highlights why integrated-strain descriptions and anticipatory models may converge. It does not settle the debate, but it does sharpen boundary conditions and generate tests that can distinguish whether and when thermal transduction pathways contribute to pacing adjustments in humans.

## Abbreviations

CG: Central governor
CNS: Central nervous system
CaMKII: Ca²⁺/calmodulin-dependent protein kinase II
EMG: Electromyography
HSP: Heat shock protein
PGC-1α: Peroxisome proliferator-activated receptor-γ coactivator-1α
RPE: Rating of perceived exertion
SR: Sarcoplasmic reticulum
T_c_: Core temperature
T_sk_: Mean skin temperature
TRP: Transient receptor potential (ion channels)
TRPA1: TRP ankyrin 1 (cold/irritant-sensitive)
TRPM2: TRP melastatin 2 (warm-sensitive)
TRPM8: TRP melastatin 8 (cool-sensitive)
TRPV1: TRP vanilloid 1 (heat/capsaicin-sensitive)
TRPV4: TRP vanilloid 4 (warm/osmo-mechanosensitive)
VO_2_: Oxygen uptake
